# Highly Sensitive Flow Cytometric Detection of Residual B-Cells After Rituximab in Anti-Neutrophil Cytoplasmic Antibodies-Associated Vasculitis Patients

**DOI:** 10.3389/fimmu.2020.566732

**Published:** 2020-12-15

**Authors:** Laura S. van Dam, Jelle M. Oskam, Sylvia W. A. Kamerling, Eline J. Arends, O. W. Bredewold, Magdalena A. Berkowska, Jacques J. M. van Dongen, Ton J. Rabelink, Cees van Kooten, Y. K. Onno Teng

**Affiliations:** ^1^Centre of Expertise for Lupus-, Vasculitis-, and Complement-Mediated Systemic Autoimmune Diseases (LuVaCs), Department of Internal Medicine, section Nephrology, Leiden University Medical Center, Leiden, Netherlands; ^2^Immunomonitoring group, Department of Immunohematology and Bloodtransfusion, Leiden University Medical Center, Leiden, Netherlands

**Keywords:** ANCA-associated vasculitis, rituximab, B-cells, immunomonitoring, glomerulonephritis, ANCA antibodies, minimal residual autoimmunity, highly sensitive flow cytometry

## Abstract

**Background:**

B-cell depletion with rituximab (RTX) is an effective treatment for anti-neutrophil cytoplasmic antibodies (ANCA)-associated vasculitis (AAV) patients. Nevertheless, relapses are frequent after RTX, often preceded by B-cell repopulation suggesting that residual autoreactive B-cells persist despite therapy. Therefore, this study aimed to identify minimal residual autoimmunity (MRA) in the B-cell compartment of AAV patients treated with RTX.

**Methods:**

EuroFlow-based highly-sensitive flow cytometry (HSFC) was employed to study B-cell and plasma cell (PC) subsets in-depth in AAV patients before and after RTX treatment. Additionally, peripheral blood mononuclear cells (PBMCs) of these RTX-treated AAV patients were cultured and *in vitro* stimulated with CpG, IL-2, and IL-21 to induce antibody-secreting cells (ASC). (ANCA)-IgG was measured in these supernatants by ELISA.

**Results:**

By employing EuroFlow-based HSFC, we detected circulating CD19^+^ B-cells at all timepoints after RTX treatment, in contrast to conventional low-sensitive flow cytometry. Pre-germinal center (Pre-GC) B-cells, memory B-cells and CD20^+^CD138^−^ plasmablasts (PBs) were rapidly and strongly reduced, while CD20^−^CD138^−^ PrePC and CD20^-^CD138^+^ mature (m)PCs were reduced slower and remained detectable. Both memory B-cells and CD20^−^ PCs remained detectable after RTX. Serum ANCA-IgG decreased significantly upon RTX. Changes in ANCA levels strongly correlated with changes in naive, switched CD27^+^ and CD27^−^ (double-negative) memory B-cells, but not with plasma cells. Lastly, we demonstrated *in vitro* ANCA production by AAV PBMCs, 24 and 48 weeks after RTX treatment reflecting MRA in the memory compartment of AAV patients.

**Conclusion:**

We demonstrated that RTX induced strong reductions in circulating B-cells, but never resulted in complete B-cell depletion. Despite strongly reduced B-cell numbers after RTX, ANCA-specific memory B-cells were still detectable in AAV patients. Thus, MRA is identifiable in AAV and can provide a potential novel approach in personalizing RTX treatment in AAV patients.

## Introduction

B-cell depletion with rituximab (RTX) is an effective treatment strategy for patients with anti-neutrophil cytoplasmic antibody (ANCA)-associated vasculitis (AAV) (1, 2) and is increasingly prescribed as induction and/or maintenance treatment (3–5). The rationale for B-cell depletion in AAV patients is the reduction of autoreactive, ANCA-producing B-cells (6). RTX, a chimeric anti-CD20 antibody, results typically in a rapid CD20^+^ B-cell depletion, while bone marrow (BM) precursors and long-lived plasma cells (PCs), which lack CD20 expression, remain unaffected (7).

Despite the success with RTX as remission-induction therapy in AAV patients (1, 2), in the RAVE-trial, one-third of the patients experienced a relapse within 18 months after RTX (8). In this trial, increases in ANCA levels did not predict relapses in either the RTX or the cyclophosphamide treatment group. Additionally, relapses were preceded by B-cell repopulation in 88% of the patients, but B-cells also returned in the two-thirds of patients who did not experience a relapse. Importantly, relapses were rare in the absence of both B-cells and ANCA (8). Nevertheless, serum ANCA-levels and B-cell numbers have been proposed as potential biomarkers that can predict these relapses and guide RTX retreatment, based on the premise that return of ANCA or B-cells are a hallmark of disease relapse (8–14). Already before the use of RTX, a meta-analysis demonstrated that rising ANCAs and persistent presence of ANCAs were associated with future relapses (positive likelihood ratio (LR+): 2.84 [1.65–4.9] and 1.97 [1.43–2.7], respectively) (15). Also, we recently demonstrated that PR3-positivity predicted future relapses in AAV patients after remission-induction therapy with RTX (16), which was also supported by another study (13). Absence of B-cell repopulation strongly predicted a relapse-free status in both PR3- and MPO-ANCA positive patients. Altogether, ANCA was found to associate with relapses in several studies, whereas the potential of circulating B-cells was not always evident. However, studies that investigated the B-cell compartment of AAV patients after RTX more in-depth found several phenotypes that were associated with relapses, e.g. incomplete B-cell depletion (17); or B-cell repopulation with relatively high number of plasmablasts (PBs) (18); switched memory B-cells (19); relatively low number of naive B-cells (17) or decreased CD5^+^ regulatory B-cells (20, 21), whereas the latter also inversely correlated with ANCA levels (22). Altogether, strongly indicating that specific subsets of (autoreactive) B-cells are involved in the pathogenesis of relapses.

Importantly, the method of analyzing B-cells, especially after RTX, and specifically the sensitivity of the method, determines the detection level of B-cell depletion and reconstitution (23). Conventional “low sensitive” flow cytometry (LSFC), applied in standard clinical care for AAV patients, is only able to detect CD19^+^ B-cells starting from 1 cell/µl. As a consequence, <1 cell/ul is then defined as “complete B-cell depletion”, without defining different B-cell subsets. In contrast, highly-sensitive flow cytometry (HSFC), originally developed to detect minimal residual disease (MRD) in hematologic malignancies (24), is able to pick up B-cells with up to 100 times more sensitivity.

In the present study, we performed an in-depth phenotypic and functional analysis of B- and plasma cells after RTX treatment in AAV patients. We employed EuroFlow-based HSFC (25, 26) after RTX and studied ANCA-specific memory B-cells *in vitro* to identify minimal residual autoimmunity (MRA).

## Materials and Methods

### Study Population

This observational prospective single cohort study was conducted at the expert center for Lupus-, Vasculitis-, and Complement-mediated systemic autoimmune diseases (LuVaCs) of the Leiden University Medical Center (LUMC) in the Netherlands. In this study, AAV patients treated with RTX were eligible and informed consent was required for study participation. The study was approved by the local medical ethics committee of the LUMC. Eleven unique AAV patients that received RTX were included in this study (**Table 1**). Seven patients received RTX as remission-induction treatment for active disease, of which 6 were included for flow cytometry studies which are shown in **Figures 2**–**4**. Additionally, four other patients and three patients from the previous group received up to 4 times maintenance treatment with 500 mg RTX every 6 months (**Supplementary Tables 1** and **2**), which were allowed to re-enter the study (**Supplementary Figure 1**). There was a total of 17 RTX maintenance treatments, of which 8 were included for flow cytometry studies (**Supplementary Table 1**). The flow cytometry data of these RTX maintenance patients were shown in the **Supplementary Figures 5** and **7**. Regarding the PBMC culture experiments, all available PBMC samples at week 0, 24, and 48 weeks after all RTX treatments in all patients were included, except one ANCA-negative patient (n = 23).

**Table 1:** Patient characteristics.

	AAV patients (n = 11)	Flow cytometry studies (n = 6)
**Demographics**		
Age	59 (33–77)	42 (33–77)
Male	6 (55%)	3 (50%)
Caucasian	11 (100%)	6 (100%)
**ANCA associated vasculitis**		
GPA	4 (36%)	2 (33%)
MPA	6 (55%)	3 (50%)
eGPA	1 (9%)	1 (17%)
**Immunology**		
**ANCA immunofluorescence**		
c-ANCA	3 (27%)	1 (17%)
p-ANCA	7 (64%)	4 (67%)
negative	1 (9%)	1 (17%)
**ELISA**		
PR3	4 (36%)	2 (50%)
MPO	6 (55%)	4 (67%)
negative	1 (9%)	1 (17%)
**Low sensitive flow cytometry** CD19+ B-cells (10^6^/L)	27 (0–311)	183.5 (1–311)
**Organ involvement**		
Constitutional symptoms	6 (55%)	3 (50%)
Mucocuteanous	3 (27%)	1 (17%)
Musculoskeletal	4 (36%)	2 (33%)
ENT	6 (55%)	2 (33%)
Renal	6 (55%)	3 (50%)
Respiratory	7 (64%)	3 (50%)
Cardiovascular	1 (9%)	1 (17%)
Central nervous system	1 (9%)	0 (0%)
Peripheral nervous system	0 (0%)	0 (0%)
Ophthalmology	3 (27%)	0 (0%)
Abdominal	3 (27%)	2 (33%)
**Disease parameters**		
BVAS	6 (0–30)	11.5 (6–30)
VDI	3 (0–12)	0.5 (0–7)
**Reason for treatment**		
New diagnosis	4 (36%)	4 (67%)
Relapse	1 (9%)	0 (0%)
Persistent disease	2 (18%)	2 (33%)
Maintenance treatment	4 (36%)	0 (0%)
**Treatment**		
Rituximab		
2× 1,000 mg	6 (55%)	6 (100%)
1× 500 mg	5 (45%)	0 (0%)
Methylprednisolone 3× 1,000 mg	4 (36%)	4 (67%)
Plasmapheresis	1 (9%)	1 (17%)
Cyclophosphamide (2× 500 mg)	1 (9%)	1 (17%)
High-dose corticosteroids	5 (45%)	5 (83%)
**Previous remission-induction treatment**		
Rituximab	5 (45%)	0 (0%)
Cyclophosphamide oral	3 (27%)	1 (17%)
Cyclophosphamide IV	2 (18%)	0 (0%)

For numerical values the median with (range) and for categorical values the frequency with percentages is shown.

### Rituximab Treatment Schedules

Treatment with RTX as remission-induction therapy was given as two times 1,000 mg RTX i.v. with a 2 week interval or as 500 mg RTX every 6 months as maintenance therapy. Directly before every RTX infusion, all patients received oral acetaminophen, clemastine i.v. and 100 mg methylprednisolone i.v. Additionally, patients were allowed to receive other immunosuppressive medication according to standardized local protocol including up to three times 1,000 mg methylprednisolone i.v. daily, followed by high dose oral corticosteroids (1 mg/kg/day, maximum 60 mg) with tapering over 3 months. Additionally, patients with severe renal and/or lung involvement were allowed to receive 500 mg cyclophosphamide i.v. and/or plasmapheresis. All patients also received prophylactic treatment with co-trimoxazole 480mg/day, proton-pump inhibition, vitamin D, calcium supplementation and, if indicated, bisphosphonates.

### Clinical and Laboratory Measurements

Clinical and laboratory data were collected at study visits just before RTX infusion and 2, 4, 12, 24, and 48 weeks after RTX treatment. Serum and PBMCs were stored for experimental studies at each study visit. Birmingham Vasculitis Activity Score (BVAS-3) was used to score disease activity during the study (27). The clinical diagnostics lab measured total immunoglobulin (Ig) levels in the sera of the patients. Standardized measurements of circulating CD19^+^ B-cells by routine flow cytometry protocols were performed at the laboratory of Hematology, with a detection limit of 1*10^6^ cells/L for CD19^+^ B-cells, further referred to as low-sensitive flow cytometry (LSFC).

### Euroflow-Based Highly-Sensitive Flow Cytometry

Peripheral blood was collected in EDTA-coated tubes at each visit. Samples were processed within 4 h after collection and analyzed by flow cytometry after bulk-lyse standard operating procedure (www.EuroFlow.org), as described before (25, 26). According to these protocols, the membranes of 20*10^6^ nucleated cells per patient sample were stained with the EuroFlow 13-color IgH-isotype B-cell tube. Per sample, 10–20*10^6^ leucocytes were measured in LSR Fortessa X-20 flow cytometer (Becton Dickinson Biosciences, San Jose, Calif). Instrument set-up and calibration were performed according to EuroFlow standard operating procedures www.EuroFlow.org (28). For data analysis, Infinicyt software version 2.0.1 (Cytognos S.L., Salamanca, Spain). was used.

Gating strategy for the identification of different major B-cell and plasma cell subsets was shown in previous publications (25, 26). B-cells were identified based on their positive CD19 expression and low forward (FSC) and sideward scatter (SSC) properties, which is typical for lymphocytes. PCs were identified by high expression for CD38 and CD27, with low expression of CD24 and CD21 in conjunction with low-to-intermediate forward light scatter and sideward light scatter. Both switched memory and PCs were subsequently subclassified based on their maturation stage and expression of distinct Ig isotypes and Ig subclasses, as previously described (25, 26). This strategy resulted in the following definitions of B-cell subsets:

Pre-germinal center B-cells, including:

CD27^−^CD38^hi^CD24^hi^CD5^+^smIgM^++^IgD^+^: immature/transitional B-cells;
CD27^−^CD38^lo^CD24^het^CD5^+^smIgM^+^IgD^++^: CD5^+^ naive B-cells;
CD27^−^CD38^lo^CD24^het^CD5^-^smIgM^+^IgD^++^: CD5^−^ naive B-cells;

Memory B-cells, including:

CD27^+^CD38^lo^CD24^het^smIgM^++^IgD^+^: unswitched memory B-cells;
CD27^+^CD38^lo^CD24^het^smIgM^−^IgD^−^: switched memory B-cells;
CD27^−^CD38^lo^CD24^het^smIgM^−^IgD^−^: switched “double negative” memory B-cells;

Plasma cells (PCs)

CD20^+^CD138^-^CD27^hi^CD38^hi^CD21^−^CD24^−^: plasmablasts;
CD20^−^CD138^-^CD27^hi^CD38^hi^CD21^−^CD24^−^: prePCs;
CD20^−^CD138^+^CD27^hi^CD38^hi^CD21^−^CD24^−^: mature PCs;

Switched memory B-cells were divided in further subsets based on CD27 expression (CD27^+^ and CD27^−^), the latter also referred to as “double negatives”. PCs were further subdivided in plasmablasts (PBs), prePCs and mPCs based on CD20 and CD138. Additionally, switched memory B-cells and PCs were further subclassified according to their Ig isotypes and Ig subclasses smIgA1^+^, smIgA2^+^, smIgG1^+^, smIgG2^+^, smIgG3^+^, and smIgG4^+^ memory B-cells and smIgM^+^-only, smIgD^+^-only, smIgA1^+^, smIgA2^+^, smIgG1^+^, smIgG2^+^, smIgG3^+^, smIgG4^+^, and smIg^−^ PCs, respectively. Absolute counts were calculated by using total leucocyte cell counts, which was measured by the clinical diagnostics lab of the LUMC. For HSFC, reliable interpretation was set at a minimum of 20 acquired events, according to the EuroFlow guidelines. Counts below 20 acquired events were considered less robust interpretation and therefore indicated in the graphs with gray areas. The absolute detection limit of HSFC (<1 acquired event was 1*10^3^ cells/ul (average of all patients and timepoints), which was indicated by the dotted lines in each graph.

### PBMC Cultures

#### Isolation

EDTA-anticoagulated peripheral blood from patients was drawn, followed by PBMC isolation with the use of Ficoll-amidotriozoate (Poli Apotheek LUMC, Leiden, The Netherlands). After washing with PBS (B. Brain Medical N.V., Oss, The Netherlands), peripheral blood mononuclear cells (PBMCs) were stored in Gibco^®^ RPMI Medium 1640 (1×) containing L-Glutamate and 25 nM HEPES (Thermo Fisher Scientific, Waltham, MA, USA), 10% Fetal Calf Serum (Bodinco BV, Alkmaar, The Netherlands), 200 U/ml Penicillin-Streptomycin (Thermo Fisher Scientific, Waltham, MA, USA) and 10% dimethyl sulfoxide (DMSO) in liquid nitrogen until further use. Buffy coats from healthy donors, who gave written informed consent for scientific use of the buffy coats, were purchased from Sanquin Blood Bank, Amsterdam, The Netherlands. PBMCs from these buffy coats were isolated following the same protocol as described above.

#### Culture

After thawing, PBMCs were cultured in Iscove’s Modified Dulbecco’s Medium (IMDM) containing L-Glutamate and 25 nM HEPES (Thermo Fisher Scientific, Waltham, MA, USA), 10% Fetal Calf Serum, 200 U/ml Penicillin-Streptomycin, and 4 nM 2-Mercaptoethanol (Sigma-Aldrich, Darmstadt, Germany). One million PBMCs were cultured in 1,000 µl IMDM per well in a sterile Costar^®^ 48-Well Flat Bottom Plate (Corning Inc, Corning, NY, USA). Up to five wells per patient or HC sample were cultured per experiment. PBMCs were polyclonally stimulated with 3.2 µg/ml class B CpG ODN (InvivoGen, Toulouse, France), 1,000 IU/ml IL-2, and 100 ng/ml IL-21 (PeproTech EC Ltd., London, United Kingdom) to induce antibody secreting cells (ASCs), adjusted from protocols that were previously published (29–31). Culture plates were incubated at 37°C, 5.0% CO_2_, and 92% RH for 10 days. After 7 days, the PBMCs from one well per sample were analyzed with flow cytometry to assess the viability of the cells and proliferation of plasma cells. After 10 days supernatants of all wells were harvested and stored at −20°C.

#### Identification of Antibody-Secreting Cells

PBMCs were analyzed with a simple standard flow cytometry panel at start and after 7 days of culturing to identify antibody-secreting cells (**Supplementary Figure 2**). Cells were stained in the dark for 30 min at 4°C with the following antibody panel: anti-CD45-PerCP, anti-CD3-FITC, anti-CD14-APC-H7 (BD Biosciences, Franklin Lakes, NJ, USA), anti-IgD-PE, anti-CD19-eFluor450, anti-CD27-PeCy7 (Thermo Fisher Scientific, Waltham, MA, USA), and anti-CD38-APC (BioLegend, San Diego, CA, USA). All washing steps were performed using PBS containing 1% BSA and 0.01% azide (FACS buffer). Stained cells were analyzed in a LSR-II flow cytometer (BD Biosciences, Franklin Lakes, NJ, USA) with FACSDiva software version 8.0.2 (BD Biosciences, Franklin Lakes, NJ, USA). FlowJo™ version 10.4.2 (FlowJo, LLC, Ashland, OR, USA) was used for gating of the different cell populations (**Supplementary Figure 2**). After live cell and single cell selection, all CD45^+^CD3^−^CD14^−^CD19^+^ cells were selected and considered B-cells. Of these B-cells, all CD27^hi^CD38^hi^ cells were considered PCs. Absolute numbers of B-cells and plasma cells were calculated based upon the absolute counts of surviving PBMCs per well in combination with CD45^+^ population in the flow cytometry.

### ELISA

In-house ELISA protocols were used to determine total IgG and IgM concentrations in the supernatants of the cultures and anti-MPO IgG and anti-PR3-IgG in sera of the patients and supernatants of the cultures. Nunc MaxiSorp™ flat-bottom ELISA plates (ThermoFisher Scientific, Waltham, MA, USA) were coated overnight at room temperature in coating buffer (0.1M Carbonate, pH 9.6) with goat anti human IgG Fc (Bethyl Laboratories, Inc, Montgomery, TX, USA), rabbit anti human IgM (Thermo Fisher Scientific, Waltham, MA, USA), human sputum myeloperoxidase (Elastin Products Company, Inc, Owensfille, MO, USA) or PR3 (isolated from neutrophils according to (32). Subsequently the plates were blocked with PBS/2% Casein for 1 h at room temperature. After washing, the plates were incubated for 1 h with sera, supernatants, and antibodies were diluted in PBS/0.05% Tween/2% Casein (Tween^®^ 20, Sigma-Aldrich, Darmstadt, Germany). All washing steps were performed with PBS/0.05% Tween. Goat-anti-Human IgG-HRP (Bethyl Laboratories, Inc, Montgomery, TX, USA) was used for the total IgG ELISA. Monoclonal anti human IgM biotin antibody (Sigma-Aldrich, Darmstadt, Germany) and poly streptavidin-HRP (Sanquin, Amsterdam, The Netherlands) was used for the IgM ELISA. ABTS containing 2 µl hydrogen peroxide 30% (Sigma-Aldrich, Darmstadt, Germany) was used as a substrate for the horseradish peroxidases (HRPs) used in the IgG and IgM ELISAs. Anti-human IgG-AP antibody (Sigma-Aldrich, Darmstadt, Germany) was used for anti-MPO and anti-PR3 IgG followed by substrate: 5 mg phosphatase substrate tablet (Sigma-Aldrich, Darmstadt, Germany) in 10 ml pNPP buffer (97 ml diethanolamine, 0.1g MgCL_2_, and 0.2 NaN_3_ in 1-L water, pH 9.8). Because MPO and PR3 are active enzymes HRP was not suitable to be used in this ELISA. ELISAs using ABTS were measured at 415 nm, while those using phosphatase substrate were measured at 405 nm.

### Statistics

All descriptive clinical data were expressed as median with [range] for numerical data or given as percentage for nominal data. All flow cytometry data were expressed as median with [range] for absolute numbers or ratio as compared to baseline. Statistical difference between two groups was determined with Mann-Whitney U test. Correlations were tested with Pearson’s correlation test (flow cytometry methods) or spearman’s correlation statistical test (changes of ANCA/B-cell subsets). All data were analyzed using SPSS statistics (IBM, New York, USA), version 23 or Prism version 8.0 (GraphPad Software, La Jolla, CA, USA).

## Results

### AAV Study Population

The characteristics of AAV patients treated with RTX were summarized in **Table 1**. Median [range] BVAS scores in patients with active disease (n = 6) that received remission-induction treatment with RTX decreased from 11.5 [6–30] to 0 [0–7] after 24 weeks and remained 0 [0–10] after 48 weeks (**Supplementary Figure 3A**). Patients that received maintenance therapy (n = 8) had a median [range] BVAS score at baseline of 4 [0–7] which decreased to 0 [0–4] after 24 weeks and to 0 [0–0] after 48 weeks (**Supplementary Figure 3B**).

### CD19+ B-Cells Were Always Detectable With HSFC After RTX in Contrast to LSFC

**Figure 1:**
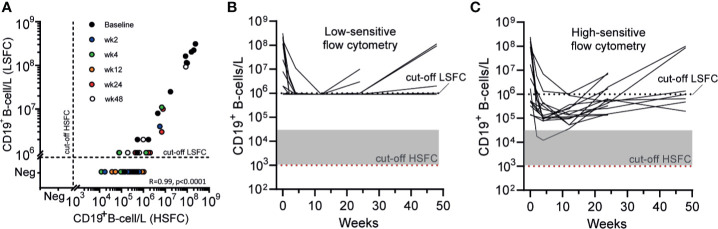
Comparing the detection of circulating CD19^+^ B-cells with LSFC versus HSFC **(A)**. Absolute numbers of circulating CD19^+^ B-cells were analyzed with HSFC (x-axis) and correlated to LSFC (y-axis) with, respectively, a detection limit of 1*10^3^ B-cells/L and 1*10^6^ B-cells/L. Each dot represents one timepoint for one patient before and 2, 4, 12, 24, or 48 weeks after RTX treatment **(B)**. Each line represents the absolute CD19^+^ B-cell count for an individual patient during RTX treatment measured with LSFC **(C)**. Each line represents the absolute CD19^+^ B-cell count for an individual patient during RTX treatment measured with HSFC. In **(B, C)**, the dotted line indicated the detection limit for LSFC (10^6^ cells/L). Gray area indicates 1–20 analyzed events. Red dotted line indicated the detection limit for HSFC (10^3^ cells/L).

Both standard clinical “low-sensitive” flow cytometry (LSFC) and EuroFlow-based HSFC (25, 26) were applied to detect B-cells in 14 AAV cases before and 2, 4, 12, 24, and 48 weeks after RTX, resulting in 68 samples. LSFC was unable to detect CD19^+^ B-cells in 43/54 samples after RTX, in contrast to HSFC which was able to detect CD19^+^ B-cells in all patients at all timepoints after RTX. **Figure 1A** illustrates the increased sensitivity of HSFC to detect B-cells up to 0.001*10^6^/L, while there is a strong positive and linear correlation between the methods (Pearson’s r = 0.99, CI [0.98–0.99]; p < 0.0001). Additionally, the number of CD19^+^ B-cells over time was shown for each individual patient after treatment with RTX as measured with LSFC (**Figure 1B**) and with HSFC (**Figure 1C**). These figures clearly emphasized the increased capability of HSFC to detect B-cells at all timepoints in contrast to LSFC.

### Residual Memory and Plasma Cells After RTX

**Figure 2:**
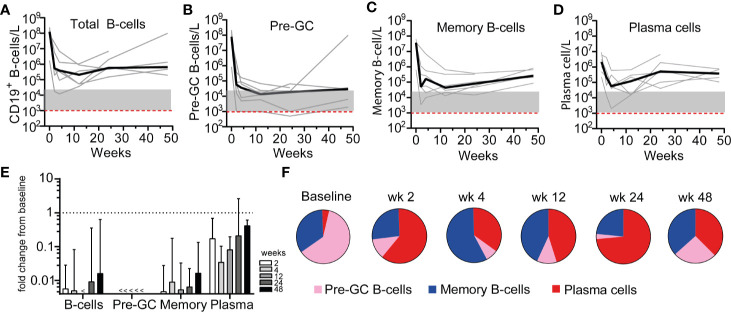
Residual memory and plasma cells after RTX. Absolute counts of **(A)** total CD19^+^ B-cells **(B)**, Pre-GC B-cells **(C)**, memory B-cells, and **(D)** plasma cells are shown for each individual patient that received remission-induction therapy with RTX (n = 6). The median is indicated by the thick black line. Red dashed line indicates the mean detection limit for HSFC. Gray area indicates 1–20 analyzed events **(E)**. Median ± IQR fold change as compared to baseline for each B-cell subset per timepoint is shown **(F)**. Mean distribution of B-cell subsets per timepoint during RTX treatment is shown.

With the ability to reliably detect low numbers of circulating B-cells with HSFC, kinetics of different B-cell and plasma cell subsets could now be assessed in AAV patients. At baseline, AAV patients with active disease (n = 6), had a median [range] of 125 [1.70–238]*10^6^ CD19^+^ B-cells/L while AAV patients in remission on maintenance RTX therapy (n = 8) had significantly less CD19^+^ B-cells/L with a median of 0.50 [0.14–17.1]*10^6^ (p = 0.003). Given the reduced B-cell numbers due to previous RTX maintenance treatments (**Supplementary Figure 4A**), the kinetics of B-cell and plasma cell subsets were studied separately in patients with active disease that received remission induction treatment with 2× 1,000 mg RTX (n = 6) (**Figure 2**). Of note, the effects of RTX as maintenance therapy (n = 8) are depicted in **Supplementary Figure 5**.

After remission-induction therapy with RTX, the nadir of circulating CD19^+^ B-cells was reached after 12 [4–24] weeks. At the nadir, concentration of CD19^+^ B-cells was 0.07 [0.01–1.05]*10^6^ B-cells/L (**Figure 2A**), corresponding to a significant decrease of −99.7% (p = 0.03) (**Figure 2E**). After the nadir, total circulating B-cells increased up to 0.58 [0.15–6.90]*10^6^ B-cells/L at 24 weeks and up to 0.65 [0.19–10.0]*10^6^ B-cells/L at 48 weeks after RTX, but remained strongly reduced as compared to baseline. As expected, RTX remission-induction treatment (2× 1,000 mg) decreased total B-cells significantly more than RTX maintenance treatment (500 mg) at all timepoints (**Supplementary Figure 4B**).

At baseline, the predominant B-cell population was pre-GC B-cells while plasma cells represented only a small subset of total B-cells (**Figure 2F**). The pre-germinal center (Pre-GC) B-cells, including immature, CD5^+^ and CD5^−^ naïve B-cell populations (**Supplementary Figure 6**) were rapidly reduced after RTX and 67% of the patients reached their nadir between 12 and 24 weeks, which was 0.01 (0.001–0.04) *10^6^ cells/L, corresponding to a significant decrease of 99.98% (**Figures 2B, E**). Additionally, the memory B-cell compartment was rapidly reduced (**Figures 2C, E**) and surprisingly also the plasma cell compartment (**Figures 2D, E**). However, the reduction of plasma cells was less profound than the memory B-cells, and the time to nadir was 12 [2–24] weeks for memory B-cells and 4 [4–12] weeks for plasma cells. The increasing numbers of total CD19^+^ B-cells 24 weeks after RTX was mainly due to an increased number of circulating plasma cells (**Figure 2F**). Because plasma cells were the least targeted population by RTX, the predominant B-cell population during RTX maintenance therapy was plasma cells (**Supplementary Figure 5F**). Of note, the absolute counts of Pre-GC B-cell subsets after maintenance therapy with RTX are shown in **Supplementary Figure 7**.

### Phenotyping Residual Memory and Plasma Cells After RTX

**Figure 3:**
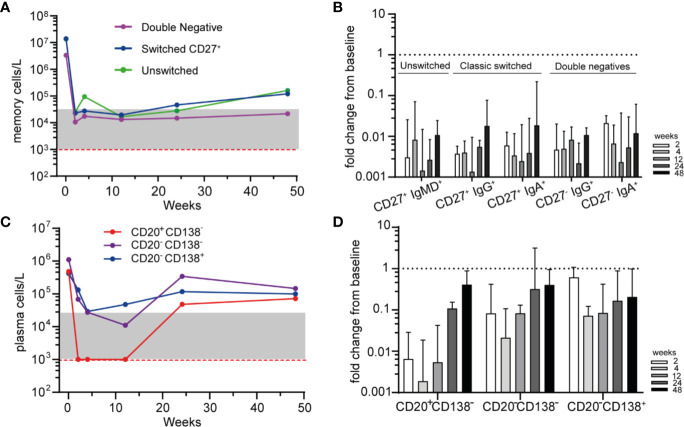
Phenotyping residual memory and plasma cells after RTX **(A)**. Median absolute counts of unswitched memory (CD27^+^IgM^+^ and/or IgD^+^), switched memory (CD27^+^IgG^+^ or IgA^+^) and double negative (DN, CD27^−^, IgG^+^, or IgA^+^) B-cells are shown for AAV patients (n = 6) after remission-induction treatment with RTX **(B)**. Median ± IQR fold change from baseline for different memory B-cell subsets are shown for each timepoint after RTX **(C)**. Median absolute counts of PBs (CD20^+^CD138^−^, red), PrePCs (CD20^−^CD138^−^, purple) and mPCs (CD20^−^CD138^+^, blue) are shown for AAV patients (n = 6) after RTX as remission-induction therapy **(D)**. Median [IQR] fold change from baseline for the subsets in A are shown for each timepoint after RTX. Red dashed line indicated the detection limit for HSFC. Gray area indicates 1–20 analyzed events.

To further study the residual memory B-cells and PCs after RTX treatment, we subsequently investigated their phenotype by HSFC. All memory subsets, including “unswitched memory B-cells” (CD27^+^ with IgM^+^ and/or IgD^+^), “switched memory B-cells” (CD27^+^ with IgA^+^ or IgG^+^) and “double negative” (DN) B-cells (CD27^−^ with IgA^+^ or IgG^+^) were rapidly and strongly reduced after RTX (**Figures 3A, B**). No significant different responses among these subgroups nor among IgG^+^ or IgA^+^ memory B-cells were detected after RTX (**Figure 3B**). Noteworthy, increasing numbers of unswitched and switched memory B-cells could be reliably detected at 48 weeks which was not the case for DN B-cells (**Figure 3A**).

Subsequently we investigated the residual plasma cell subsets based on their maturation stage and their expression of Ig isotypes after RTX (**Figure 3C**). Not unexpectedly, CD20^+^CD138^−^ plasmablasts (PBs) were rapidly undetectable after RTX, while CD20^−^CD138^−^ “Pre-PCs” and CD20^−^CD138^+^ mature PCs (mPCs) were reduced but remained detectable at all timepoints (**Figures 3C, D**). The distribution of IgA^+^, IgG^+^, and IgM^+^ plasma cell subsets was shown in **Supplementary Figure 8**. At baseline and after RTX, the most prevalent Ig subtype on PBs, PrePCs and mPCs was IgA.

### Changes of Circulating ANCAs Associated With Changes of Naive and Memory B-Cells But Not With Plasma Cells After RTX

**Figure 4:**
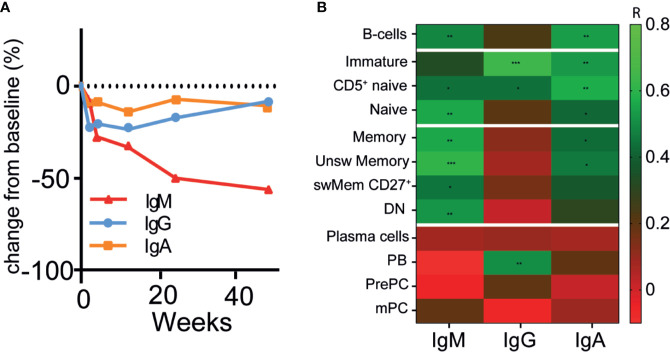
Changes of circulating immunoglobulins were not reflected by plasma cell kinetics after RTX **(A)**. Median percentage change in total immunoglobulin -M (red line), G (blue line) and -A (orange line) levels after RTX as remission-induction treatment over time (n = 6) **(B)**. Heatmap of spearman correlations of the change in IgG, IgM, and IgA with the change in different B-cell subsets as compared to baseline for all timepoints (n = 6). Gradients indicates Spearman’s R. *p < 0.05, **p < 0.01. ***p < 0.001.

Because HSFC provided measurable, longitudinal changes of B- and plasma cell subsets in AAV patients after RTX, we investigated whether these changes reflected simultaneous changes of circulating Ig and ANCA levels. The absolute serum levels of IgM, IgG and IgA after RTX in patients that received RTX as remission-induction treatment are shown in **Supplementary Figure 9**. IgM levels decreased gradually without recovery after RTX in most patients to a median decrease of −54% [−61; +37] (p = 0.31) after 48 weeks (**Figure 4A**). Total serum IgG levels reached their nadir after 4 weeks, corresponding to a median decrease of −21% [−53; −13] (p = 0.03) (**Figure 4A**). Subsequently, IgG levels gradually recovered back to baseline levels at 48 weeks. Total serum IgA levels remained largely unaffected by RTX treatment, but decreased to a median change of −11% [−44; +40] as compared to baseline (**Figure 4A**). In AAV patients that received RTX maintenance treatment circulating Ig levels did not change much (**Supplementary Figure 10**).

Surprisingly, the changes in serum IgM, -G, and -A levels were not associated with the changes in the plasma cell compartment (r < 0.2) (**Figure 4B**), which was also the case for patients that received maintenance treatment with RTX (**Supplementary Figure 10E**). Moreover, changes in IgM levels were strongly associated with changes in unswitched memory B-cells (r = 0.61, p = 0.001) and naive B-cells (r = 0.56, p = 0.002) (**Figure 4B**). The changes in IgG levels were strongly associated with changes of the immature B-cell compartment (r = 0.64, p = 0.0001), CD5^+^ naive B-cells (r = 0.44, p = 0.02), and PBs (r = 0.51, p = 0.006). Changes in IgA levels were associated with changes of CD5^+^ naïve B-cells (r = 0.57, p = 0.002) and unswitched memory B-cells (r = 0.46, p = 0.01).

**Figure 5:**
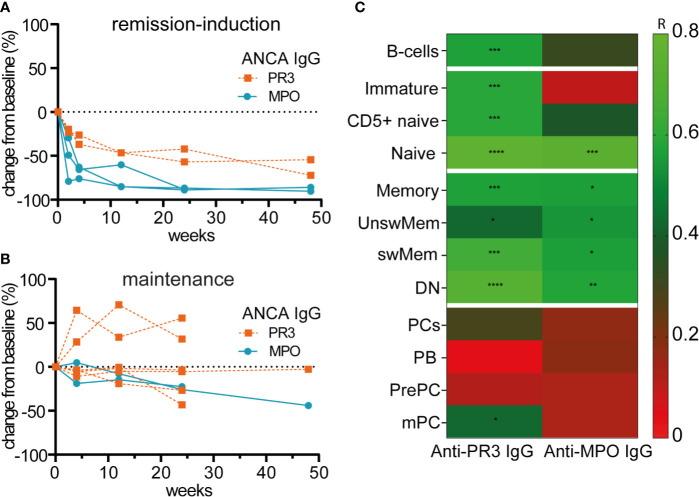
Changes of circulating ANCAs associated with changes of naive and memory B-cells but not with plasma cells after RTX **(A)**. Individual percentage change as compared to baseline of anti-PR3 and anti-MPO IgG serum levels after remission-induction with RTX treatment (n = 5) **(B)**. Individual percentage change as compared to baseline of anti-PR3 and anti-MPO IgG serum levels is shown for each patient that received RTX as maintenance treatment (n = 8) **(C)**. Heatmap of the Spearman correlations of changes in anti-MPO IgG (n = 6) and anti-PR3 IgG (n = 8) with changes in B-cell subsets as compared to baseline for all timepoints (n = 14). Gradients indicates Spearman’s R. *p < 0.05, **p < 0.01, ***p < 0.001, ****p < 0.0001.

**Figure 5** illustrates the relative changes from baseline of serum anti-PR3-IgG and anti-MPO-IgG in patients treated with RTX as remission-induction and as maintenance treatment. Absolute values are shown in **Supplementary Figure 11**. All ANCA IgG, against PR3 or MPO significantly decreased for all patients that received remission-induction treatment with RTX (**Figure 5A**). ANCA IgG was less affected during maintenance treatment with RTX (**Figure 5B**). Altogether, changes in both anti-PR3 and anti-MPO IgG autoantibodies were strongly associated with changes of naive B-cells (resp. r = 0.76, p = 0.0001 and r = 0.73, p = 0.0001) and DN memory B-cells (resp. r = 0.72, p = 0.0001 and r = 0.58, p = 0.008) (**Figure 5C**). Surprisingly, but in line with the total Ig data, changes in ANCA did not associate with changes of plasma cell subsets except a weak association of changes in anti-PR3 IgG and mPCs (r = 0.43, p = 0.02) (**Figure 5C**). Of interest, changes in MPO-ANCA IgM and IgA autoantibody levels demonstrated similar dynamics and correlations as MPO-ANCA IgG, which were significant correlations with the changes in naive B-cells (resp. r = 0.76, p = 0.0001 and r = 0.75, p = 0.0001) and DN memory B-cells (resp. r = 0.58, p = 0.008 and r = 0.55, p = 0.01), and no association with any of the plasma cell subsets (data not shown).

### Minimal Residual Autoimmunity: Presence of ANCA-Specific Memory B-Cells After RTX

**Figure 6:**
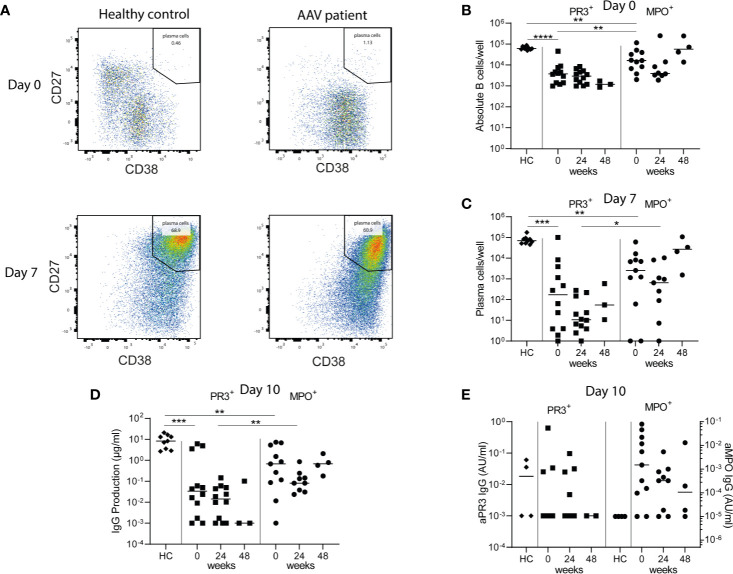
Minimal residual autoimmunity after RTX: presence of ANCA-specific memory B-cells. 1*10^6^ PBMCs/well from healthy controls (HCs) and AAV patients before, 24 and 48 weeks after RTX treatment, were stimulated for 10 days with CpG ODN class B, IL-2, and IL-21 to induce antibody-secreting cells (ASCs) in a 48-well plate **(A)**. Representative bivariate dot plots of ASCs at day 0 and day 7 of PBMC cultures demonstrated the induction of CD27^++^CD38^++^ ASCs 7 days after polyclonal stimulation of PBMCs from a HC and an AAV patient (MPO-ANCA) **(B)**. Absolute counts of total CD19^+^ B-cells were shown for each individual at baseline of the cultures (day 0) **(C)**. Absolute counts of induced ASCs per well were shown for each individual after 7 days of culturing **(D)**. Total IgG production was measured in the supernatants of each well after 10 days of culturing. Here the median of 5 wells is shown per individual **(E)**. Total ANCA-IgG production was measured in the supernatants of each well after 10 days of culturing. Anti-PR3 IgG and anti-MPO IgG are, respectively, shown on the left and right y-axis. Each dot represents the median of 5 wells for each individual sample. *p < 0.05, **p < 0.01, ***p < 0.001, ****p < 0.0001.

To ultimately demonstrate whether the observed residual memory B-cells with HSFC harbored ANCA-specific B-cells, we investigated *in vitro* ANCA production before and after RTX treatment. To do so, total PBMCs from AAV patients were polyclonally stimulated to induce antibody-secreting cells (ASCs) and subsequently (ANCA) IgG was measured in their supernatants as a reflection of ANCA-specific memory B-cells (**Figure 6A**). At baseline of the PBMC culture, the number of CD19^+^ B-cells for HCs was 61[56-74]*10^3^/well out of 1*10^6^ PBMCs/well, corresponding to normal range references values of HCs (~6%) (33) (**Figure 6B**). Both PR3- and MPO-ANCA AAV patient samples had significantly lower starting numbers of B-cells in the culture as compared to HCs, possibly due to previous immunosuppressive treatment (**Figure 6B**).

Polyclonal stimulation of PBMCs from HCs resulted in a median [range] of 70 [45–170]*10^3^ CD27^++^CD38^++^ ASCs per well after 7 days (**Figure 6C**). Polyclonal stimulation of PBMCs from PR3-ANCA and MPO-ANCA AAV patient samples before RTX treatment resulted in 0.17 [0.001–100]*10^3^ and 2.6 [1–61]*10^3^ ASCs (**Figure 6C**). After 10 days of culturing, PBMCs from HCs produced a median of 8.3 [2.7–20.7] µg/ml IgG, while 0.03 [0–6.1] µg/ml IgG by PR3-ANCA patient samples and 0.7 [0–7.3] µg/ml IgG by MPO-ANCA patient samples was detected (**Figure 6D**). Importantly, relevant IgG levels could also be detected at 24 and 48 weeks after RTX. Most importantly, ANCA IgG production was detectable in 3/12 PR3-ANCA and 9/11 MPO-ANCA patient samples at baseline. At 24 weeks after RTX, ANCA IgG production was detectable in 4/12 PR3-ANCA and 7/9 MPO-ANCA patient samples and at 48 weeks after RTX in 0/2 PR3-ANCA and 3/4 MPO-ANCA patient samples (**Figure 6E**). Altogether, these PBMC cultures demonstrated that both PR3-ANCA and MPO-ANCA patients had residual ANCA-specific memory B-cells after RTX.

## Discussion

This study aimed to investigate MRA in the B-cell compartment of AAV patients after RTX. We demonstrated that despite significant reductions in circulating B-cell numbers after RTX, B-cells always remained detectable when employing Euroflow-based HSFC. Residual B-cells after RTX were predominantly memory B-cells and CD20^−^ plasma cells. Longitudinal changes in the plasma cell compartment were not associated with changes in serum ANCA levels. Within residual B-cells after RTX, we demonstrated the presence of ANCA-specific memory indicative of MRA in AAV patients. RTX is an effective treatment for AAV patients which is increasingly used both for remission-induction and maintenance treatment. Nevertheless, relapses are common after RTX, which are not always reliably predicted through immunomonitoring of serum ANCAs and/or total circulating CD19^+^ B-cell numbers. Still, much evidence points toward the role of B-cells in the pathogenesis of relapses in AAV patients.

Euroflow-based HSFC always detected circulating CD19^+^ B-cells after RTX in any patient, demonstrating that B-cell depletion after RTX is never absolute and that it depends on the sensitivity of the flow cytometry method, which is at best 1*10^6^ B-cells/L in routine clinical practice and large clinical studies (5, 34). However, it was previously demonstrated that after RTX, AAV patients with residual B-cells, either defined as ≥1*10^6^ B-cells/L (16) or ≥0.1*10^6^ B-cells/L (17) had worse clinical responses. It is also well described that patients can relapse with B-cells below the conventional threshold of flow cytometry (5, 35, 36). Additionally, the return of B-cells after RTX has also been recognized as a risk factor for relapse (16, 37), and successfully used as a biomarker to reduce RTX infusions (5). Moreover, different studies have shown that specific B-cell populations have a distinct role in AAV disease. The repopulation of naive B-cells after RTX at 6 months was associated with a reduced risk of relapse (17). Also regulatory B-cells (Breg) have been described as a key B-cell subgroup responsible for maintaining self-tolerance (38). Indeed, these Bregs, present among CD5^+^ B-cells inversely correlated with disease activity in AAV patients after RTX (20, 21). Recently, CD27^+^CD38^++^ plasma cells were increased in patients at baseline that relapsed in the future (18). Altogether, these studies support the implementation of HSFC as an immunomonitoring tool in AAV patients. With HSFC, potential new biomarkers for RTX can be identified which are more closely associated to its B-cell depleting mechanism of action than, for example, serum ANCA levels. Therefore, further studies are needed to investigate the added value of HSFC to predict treatment efficacy and to guide personalized treatment strategies in AAV patients.

An interesting finding in our study was that the changes in ANCA levels closely related to reductions in naive, switched memory and DN B cells, but not plasma cells. There are reports that naive B-cells can contain antigen-experienced cells, including B-cells recognizing RhD and tetanus (39). Also, PR3^+^ B-cells were detected in the naive B-cell compartment in both HCs and PR3^+^ AAV patients previously (40). In this study, the feasibility of detecting PR3-specific B-cells by HSFC was demonstrated (40). There was no correlation between PR3-ANCA titer and % PR3^+^ B-cells, while PR3^+^ B-cells were phenotypically enriched in the switched memory B-cell and PB subsets. Whether PR3-specific B-cells can be detected within low B-cell levels after RTX remains to be demonstrated. We demonstrated here the proof-of-concept that indeed ANCA-specific memory B-cells persist after RTX.

Moreover the correlation of unswitched memory B-cells and serum IgM levels were consistent with a previous study (41). In this study marginal zone (MZ)-like B-cells (CD19^+^CD27^+^IgD^+^IgM^high^, comparable with unswitched memory B-cells in our study), negatively correlated with MPO- and PR3-ANCA levels, whereas we found a positive significant correlation of the changes in MPO- and PR3-ANCA levels with the changes in unswitched memory B-cells. Possibly this is due by the setting of the study: we studied the changes in ANCAs and B-cells after RTX treatment in our study, whereas Appelgren et al. studied the correlations of absolute ANCA levels and MZ-like B-cells in active treatment naïve patients.

Another interesting finding in this study employing HSFC was the observation that RTX not only reduced CD20^+^ B cell subsets but was also associated with significant reductions in CD20^−^ prePCs and mPCs. This observation strongly suggested that these reductions were an indirect effect of RTX most likely explained by the strong decrease of their precursors and/or migration of CD20^−^ PCs out of the circulation while entry of their precursors lacked.

The present study had some limitations. First, the study was designed as a proof-of-concept study to investigate the potential use of HSFC after RTX treatment. As such, the number of patients included in this study was too low to assess the value of HSFC as biomarker in relation to disease activity or relapses. To achieve this, the study population should also have been treated in a homogenous, standardized RTX-based treatment strategy. Secondly, it needs to be acknowledged that RTX is known to mask the CD20 epitope in competition with diagnostic antibodies used for flow cytometry (42). As such, CD20 expression up to 12 weeks after RTX needs to interpret with caution, notably the CD20^+^CD138^−^ PB and CD20^−^CD138^−^ PrePCs in our study. Thirdly, when investigating *in vitro* cultures of PBMC, the detection of ANCA in supernatants depends upon the frequency of ANCA-specific B-cells. Therefore, because B-cell numbers are low after RTX, we cannot firmly conclude that *in vitro* undetectable ANCA levels in the supernatant actually relate to the successful eradication of ANCA-specific B-cells.

In conclusion, this study demonstrates the presence of residual ANCA-specific memory B-cells in AAV patients after RTX. Moreover, EuroFlow-based HSFC demonstrated that RTX strongly reduced circulating B-cells but never fully depleted them. Residual B-cells consisted predominantly of memory B-cells and plasma cells. Changes in ANCA levels associated predominantly with changes in naive, switched or DN memory B-cells but not plasma cells. Altogether, we provide evidence for MRA in the memory B-cell compartment after RTX in AAV. Further studies are warranted to better assess MRA and its association with disease activity and relapses in AAV patients.

## Data Availability Statement

The raw data supporting the conclusions of this article will be made available by the authors, without undue reservation.

## Ethics Statement

The studies involving human participants were reviewed and approved by CME LUMC LEIDEN. The patients/participants provided their written informed consent to participate in this study.

## Author Contributions

LD, JO, SK, and EJA carried out the experiments, LD, MB, and JO analyzed the data. LD made the figures. LD, OWB, MB, JD, CK, and YT designed the study. All authors contributed to the article and approved the submitted version.

## Funding

The Dutch Kidney Foundation (KJPB12.028 & 17OKG04) and the Netherlands Organization for Scientific Research. FOREUM – SLE project.

## Conflict of Interest

The authors declare that the research was conducted in the absence of any commercial or financial relationships that could be construed as a potential conflict of interest.
