# Supplementary material for: Highly Sensitive Flow Cytometric Detection of Residual B-Cells After Rituximab in Anti-Neutrophil Cytoplasmic Antibodies-Associated Vasculitis Patients
Source: Front Immunol. 2020 Dec 15;11:566732. doi: 10.3389/fimmu.2020.566732 (PMC7770159; doi:10.3389/fimmu.2020.566732)
Supplement: Supplementary file 1 [file DataSheet_1.docx]

**Supplementary figures**

**Supplemental material Table of contents**

| **Figure** | **Title** |
| --- | --- |
| Supplementary Table 1 | Overview of patients sampling |
| Supplementary Table 2 | Patient characteristics receiving maintenance RTX |
| Supplementary Figure 1 | RTX treatment schedule |
| Supplementary Figure 2 | Gating strategy for identification of B and plasma cells before and after PBMC cultures |
| Supplementary Figure 3 | Disease activity in AAV patients after RTX |
| Supplementary Figure 4 | CD19^+^ B-cells in AAV patients treated with RTX as remission-induction (RI) treatment versus maintenance (Main) treatment |
| Supplementary Figure 5 | Kinetics of different B-cells subsets after maintenance therapy with RTX |
| Supplementary Figure 6 | Kinetics of pre-germinal center B-cell subsets after remission-induction therapy with RTX. |
| Supplementary Figure 7 | Kinetics of pre-germinal center B-cell subsets after maintenance therapy with RTX. |
| Supplementary Figure 8 | Changes of IgM^+^D^+^, IgG^+^- or IgA^+^-specific memory B-cells and IgG^+^, IgA^+^, IgM^+^ or Ig- plasma cell subsets after RTX |
| Supplementary Figure 9 | Total serum immunoglobulin levels after remission induction treatment with RTX |
| Supplementary Figure 10 | Changes of total serum immunoglobulin levels during maintenance treatment with RTX and correlations with changes in B-cell subsets after RTX |
| Supplementary Figure 11 | Absolute serum values of anti-PR3 and anti-MPO IgG of AAV patients treated with RTX as remission induction and as maintenance treatment |

**Supplementary table 1. Overview of patients sampling**

| **Patients** | **RTX treatments** | | | | |
| --- | --- | --- | --- | --- | --- |
|  | remission-induction | maintenance 1 | maintenance 2 | maintenance 3 | maintenance 4 |
| **MORIA 1** | FC | x | x | x | x |
| **MORIA 2** | F | x | x | x | x |
| **MORIA 3** | X | FC | C | FC | FC |
| **MORIA 4** | X | x | FC | C | FC |
| **MORIA 5** | FC | C | x | x | x |
| **MORIA 6** | X | x | C | C | C |
| **MORIA 7** | FC | FC | FC | FC | x |
| **MORIA 8** | C | C | x | x | x |
| **MORIA 9** | X | x | x | C | C |
| **MORIA 10** | FC | x | x | x | x |
| **MORIA 11** | FC | x | x | x | x |

F=HSFC, C=PBMC cultures, x=not applicable

**Supplementary table 2. Patient characteristics receiving maintenance RTX**

|  | AAV patients (n=7) |
| --- | --- |
| **Demographics** |  |
| Age | 59 (39-72) |
| Male | 5 (71%) |
| Caucasian | 7 (100%) |
| **ANCA associated vasculitis** |  |
| GPA | 3 (43%) |
| MPA | 4 (57%) |
| eGPA | 0 (0%) |
| **Immunology** |  |
| **ANCA immunofluoresence** |  |
| c-ANCA | 2 (29%) |
| p-ANCA | 5 (71%) |
| negative | 0 (0%) |
| **ELISA** |  |
| PR3 | 3 (43%) |
| MPO | 4 (57%) |
| Negative | 0 (0%) |
| CD19+ B-cells (10^6^/L)  low sensitive flow cytometry | 2 (0-27) |
| **Organ involvement** |  |
| Constitutional symptoms | 5 (71%) |
| Mucocuteanous | 2 (29%) |
| Musculoskeletal | 3 (43%) |
| ENT | 5 (71%) |
| Renal | 4 (57%) |
| Respiratory | 5 (71%) |
| Cardiovascular | 1 (14%) |
| Central nervous system | 1 (14%) |
| Peripheral nervous system | 0 (0%) |
| Opthalmology | 3 (43%) |
| Abdominal | 2 (29%) |
| **Disease parameters** |  |
| BVAS | 2 (0-11) |
| VDI | 5 (0-12) |
| **Reason for treatment** |  |
| New diagnosis | 0 (0%) |
| Relapse | 0 (0%) |
| Persistent disease | 0 (0%) |
| Maintenance treatment | 7 (100%) |
| **Treatment** |  |
| Rituximab |  |
| 1x500mg | 7 (100%) |
| **Previous remission-induction treatment** |  |
| Rituximab | 7 (100%) |
| Cyclophosphamide oral | 2 (29%) |
| Cyclophosphamide IV | 2 (29%) |

**
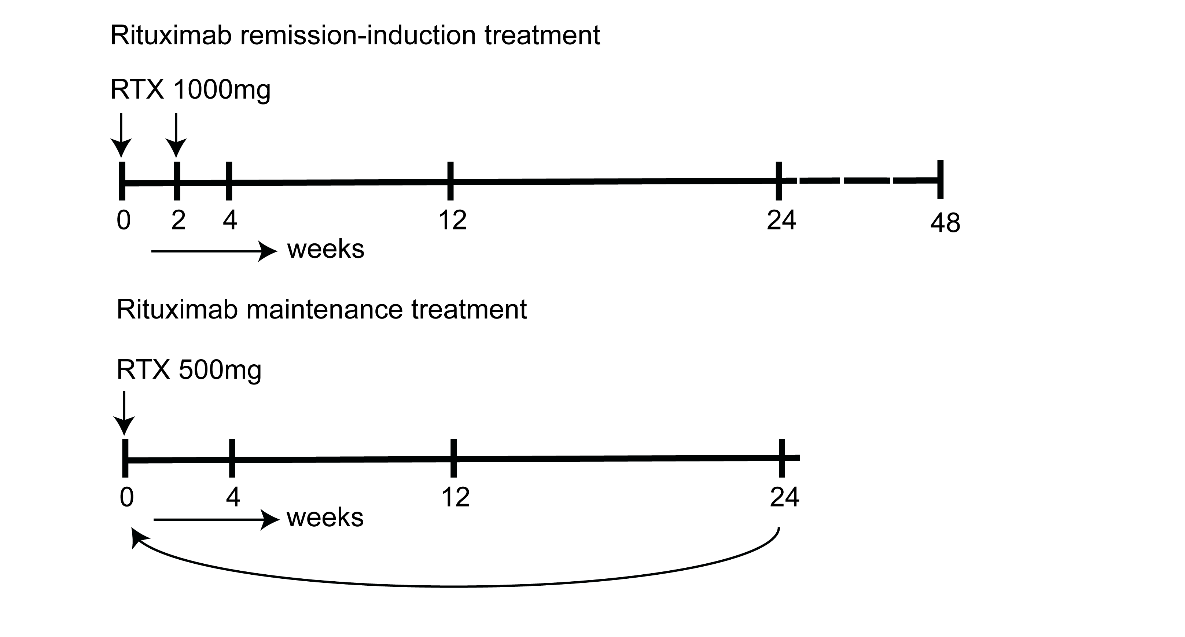
Supplementary figure 1.** RTX treatment schedule. Patients receiving 1000mg rituximab remission-induction treatment at week 0 and week 2 were sampled at week 0, week 2, week 4, week 12, week 24 and week 48. Patients receiving maintenance RTX treatment received 500mg at week 0 and were sampled week 0, week 4, week 12 and week 24.

**
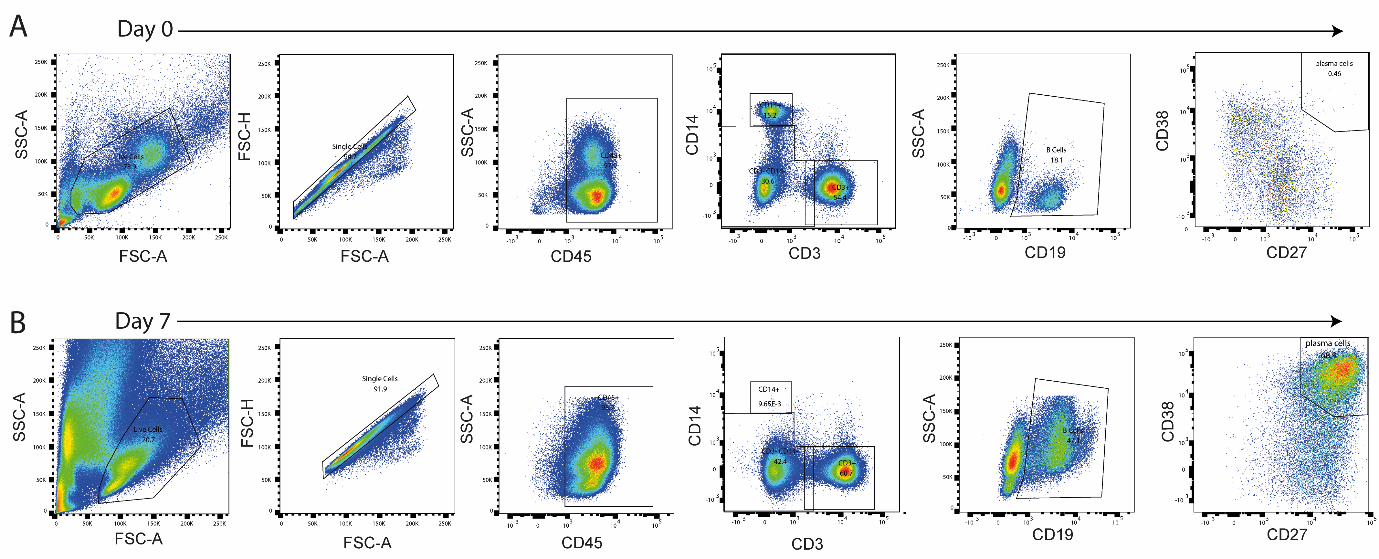
**

**Supplementary figure 2. Gating strategy for identification of B and plasma cells before and after PBMC cultures.** A) Representative bivariate dot plots show the gating strategy of PBMCs with flow cytometry at baseline to determine the starting values of B-cells in the PBMC cultures. B) Representative bivariate dot plots show the gating strategy 7 days after polyclonal stimulation of PBMCs to identify plasma cells with flow cytometry. For both strategies the following populations were selected; (1) live cells, (2) single cells, (3) CD45^+^ cells, (4) CD3^-^ and CD14^-^ cells, (5) CD19^+^ B-cells and (6) CD27^++^ CD38^++^ antibody-secreting cells (ASCs).

**
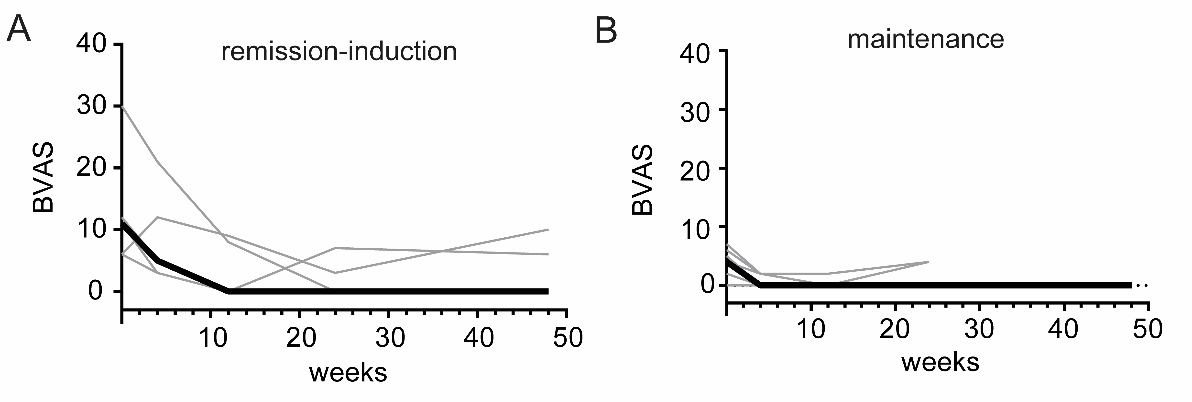
**

**Supplementary figure 3. Disease activity in AAV patients after RTX.** A) Individual BVAS scores over time of AAV patients with active disease that were treated with RTX as remission-induction treatment (n=6). B) Individual BVAS scores over time of AAV patients in remission that were treated with RTX as maintenance treatment (n=8). Median is indicated by the thick black line.

**
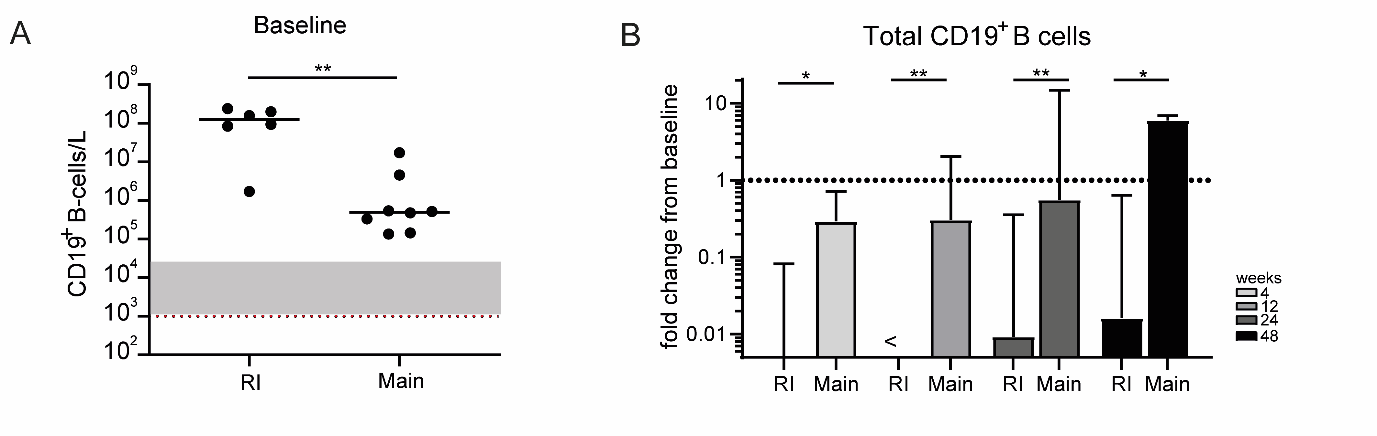
Supplementary figure 4. CD19^+^ B-cells in AAV patients treated with RTX as remission-induction (RI) treatment versus maintenance (Main) treatment.** A) Absolute number of CD19^+^ B-cell/L was shown for AAV patients with active disease before start of remission-induction (RI) treatment with RTX (2x1000mg) and compared to absolute number of CD19^+^ B-cell/L in patients in remission treated with RTX as maintenance (Main) treatment (500mg). Red dashed line indicated the mean detection limit for HSFC. Grey area indicates 1-20 analyzed events. B) Fold changes of absolute number of CD19^+^ B-cell/L as compared to baseline at 4, 12, 24 and 48 weeks after RTX were demonstrated and compared between RI and Main group. Mann-Whitney U test was used to test the statistical differences between two groups. *p<0.05, **p<0.01.

**
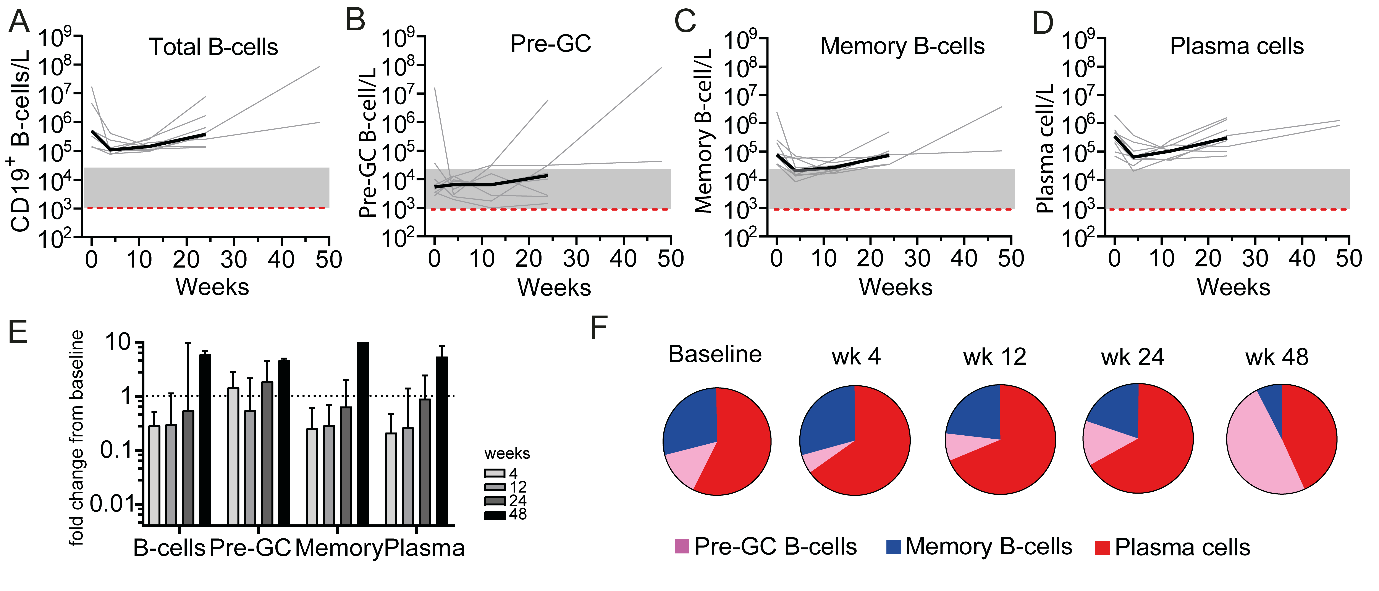
**

**Supplementary figure 5.** **Kinetics of different B-cells subsets after maintenance therapy with RTX.** Absolute counts of A) total CD19^+^ B-cells, B) Pre-GC B-cells C) memory B-cells and D) plasma cells are shown for each patient that received RTX as maintenance therapy (individual lines) (n=8). The median is indicated by the thick black line. Red dashed line indicates the mean detection limit for HSFC. Grey area indicates 1-20 analyzed events. E) Median±IQR decrease in percentage as compared to baseline for each B-cell subset (A-D) per timepoint is shown (n=8). F) Mean distribution of B-cell subsets per timepoint during RTX treatment is shown (n=8).

**
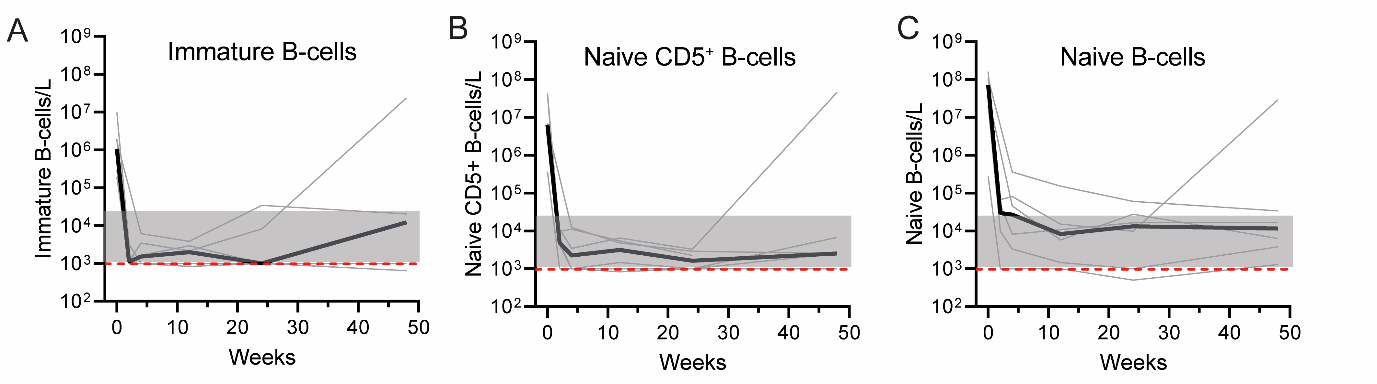
**

**Supplementary figure 6. Kinetics of pre-germinal center B-cell subsets after remission-induction therapy with RTX.** Absolute counts of A) Immature B-cells, B) Naive CD5^+^ B-cells and C) naive B-cells are shown for each patient that received remission-induction therapy with RTX (individual grey lines) (n=6). The median is indicated by the thick black line. Red dashed line indicated the mean detection limit for HSFC. Grey area indicates 1-20 analyzed events.

**
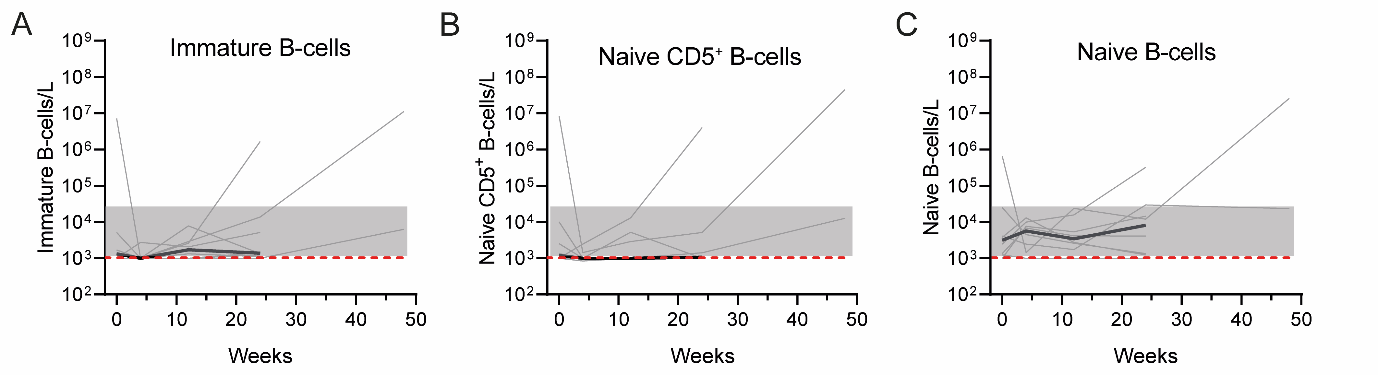
**

**Supplementary figure 7. Kinetics of pre-germinal center B-cell subsets after maintenance therapy with RTX.** Absolute counts of A) Immature B-cells, B) Naive CD5^+^ B-cells and C) naive B-cells are shown for each patient that received RTX as maintenance therapy (individual grey lines) (n=8). The median is indicated by the thick black line. Red dashed line indicated the mean detection limit for HSFC. Grey area indicates 1-20 analyzed events.

**
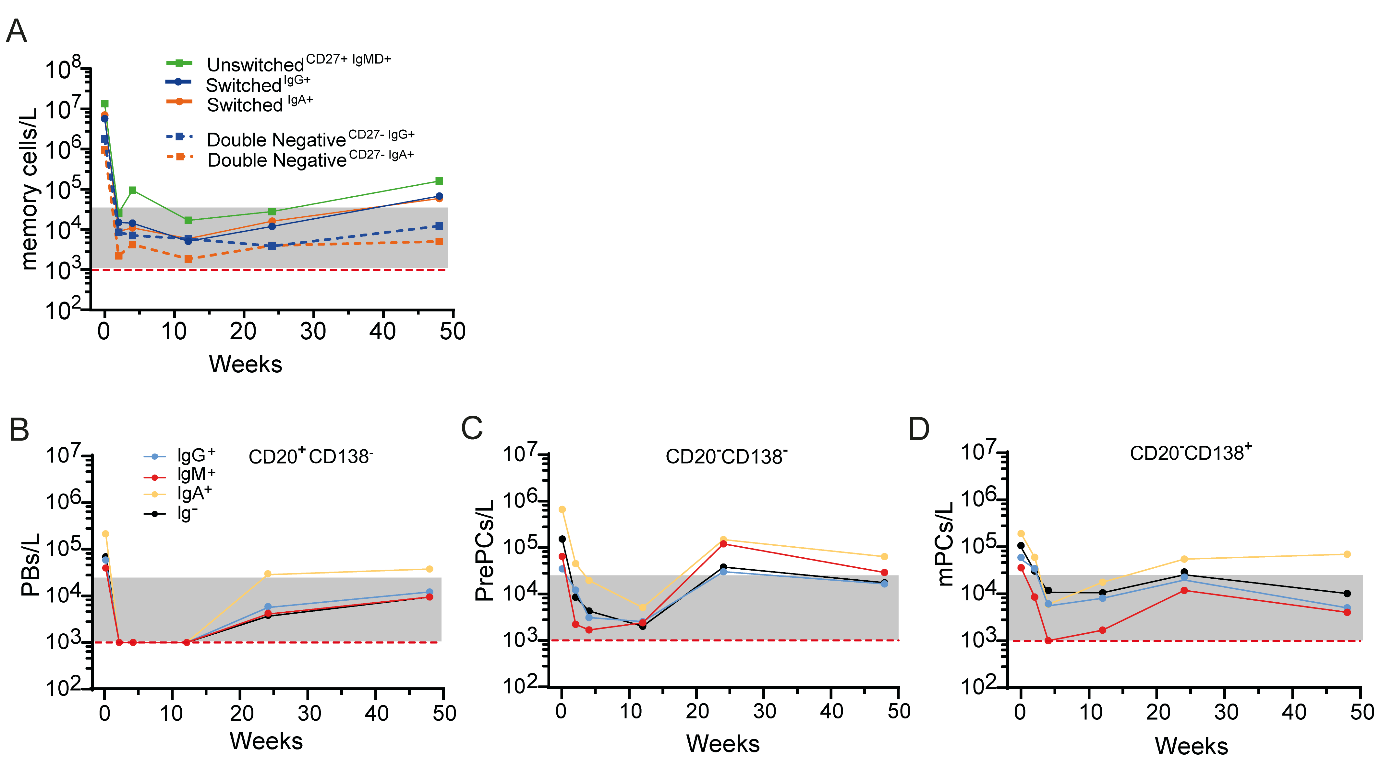
**

**Supplementary figure 8. Changes of IgM^+^D^+^, IgG^+^- or IgA^+^-specific memory B-cells and IgG^+^, IgA^+^, IgM^+^ or Ig- plasma cell subsets after RTX.** A) Median absolute counts of IgM^+^D^+^, IgG^+^- or IgA^+^-specific memory B-cells are shown for AAV patients (n=6) after RTX as remission-induction therapy. B) Median absolute counts of IgG^+^, IgM^+^, IgA^+^ and Ig^-^ plasma cells are shown for AAV patients (n=6) after RTX as remission-induction therapy. Red dashed line indicated the detection limit for HSFC. Grey area indicates 1-20 analyzed events.

**
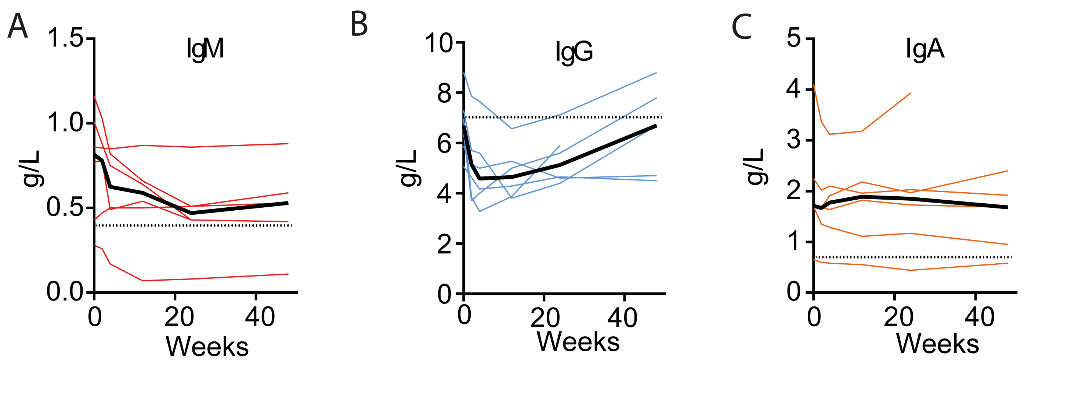
**

**Supplementary figure 9. Total serum immunoglobulin levels after remission induction treatment with RTX.** A-C) Total serum (A) IgG, (B) IgM, and (C) IgA g/L levels in each patient (n=6) (individual lines) during RTX as remission-induction treatment. The median is indicated by the thick black line and the normal range is indicated by the dotted line.

**
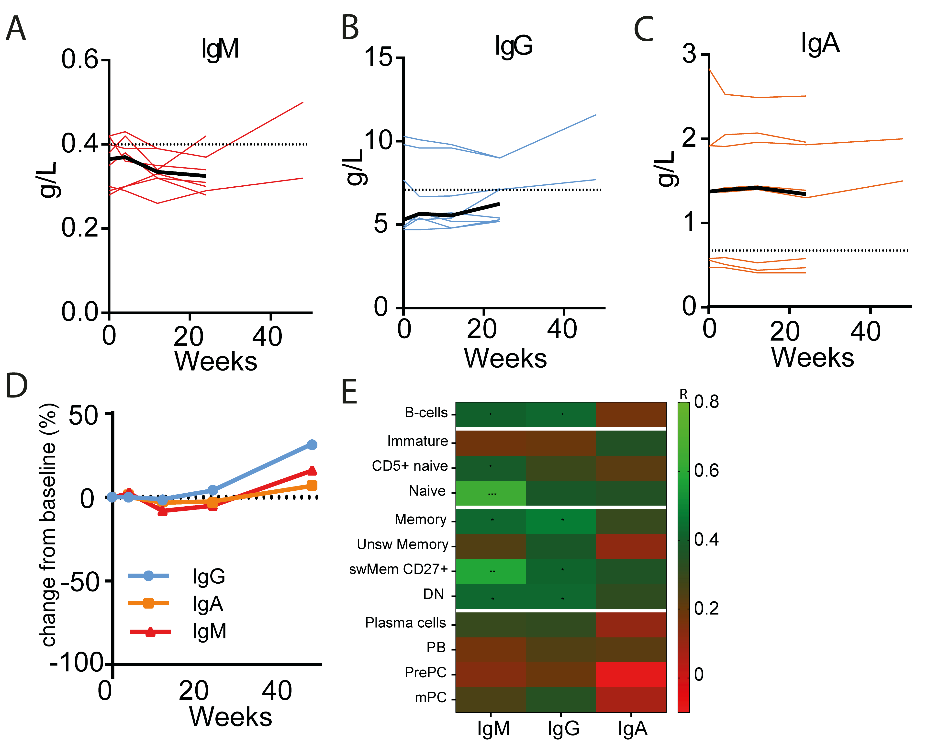
**

**Supplementary figure 10. Changes of total serum immunoglobulin levels during maintenance treatment with RTX and correlations with changes in B-cell subsets after RTX.** A-C) Total serum (A) IgG, (B) IgM, and (C) IgA g/L levels in each patient (n=8) (individual lines) during RTX maintenance treatment. The median is indicated by the thick black line and the normal range is indicated by the dotted line.. D) Median percentage change as compared to baseline for total immunoglobulin levels after maintenance treatment with RTX over time (n=8). E) Heatmap of spearman correlations of the changes in longitudinal serum level IgG, IgM and IgA with the changes in B-cell subset absolute numbers as compared to baseline for all timepoints is shown (n=8). Gradients indicates Spearman’s R. *p<0.05, **p<0.01. ***<0.001.


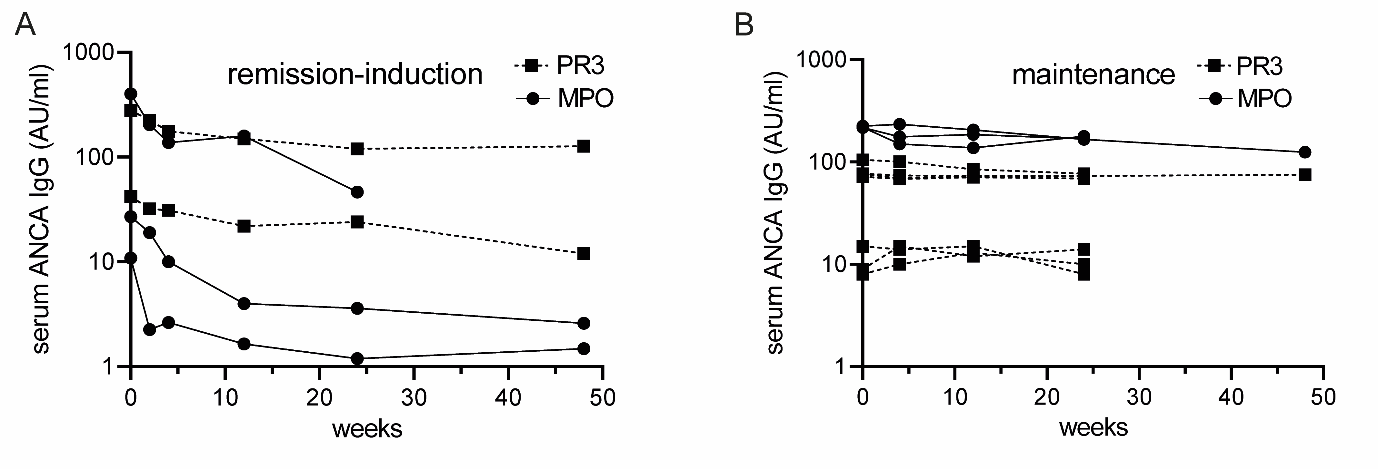


**Figure 11. Absolute serum values of anti-PR3 and anti-MPO IgG of AAV patients treated with RTX as remission induction and as maintenance treatment.** A) Individual absolute serum values of anti-PR3 and anti-MPO IgG were shown for AAV patients during RTX as remission-induction treatment (n=5). B) Individual absolute serum values of anti-PR3 and anti-MPO IgG were shown for AAV patients during RTX as maintenance treatment (n=8).
